# Association Between Eating Attitudes and Dental Caries Experience in Young Adults Attending a Private Dental Practice: A Cross-Sectional Study

**DOI:** 10.3390/nu18193273

**Published:** 2026-10-05

**Authors:** Adina Oana Armencia, Andrei Nicolau, Roxana Ionela Vasluianu, Alexandra Boloș, Carina Ana-Maria Balcos, Nicoleta Ioanid, Gabriel Condurache, Anca Mihaela Vitalariu, Irina Gradinaru, Georgiana Macovei

**Affiliations:** 1 Grigore T. Popa University of Medicine and Pharmacy Iasi, 700115 Iasi, Romania; adina.armencia@umfiasi.ro (A.O.A.); nicolau.andrei@umfiasi.ro (A.N.); alexandra.bolos@umfiasi.ro (A.B.); carina.balcos@umfiasi.ro (C.A.-M.B.); nicoleta.ioanid@umfiasi.ro (N.I.); anca.vitalariu@umfiasi.ro (A.M.V.); irina.gradinaru@umfiasi.ro (I.G.); georgiana.macovei@umfiasi.ro (G.M.); 2 Private Dental Practice, 700106 Iași, Romania; geany_rbc@yahoo.com

**Keywords:** feeding behavior, dental caries, health behavior, body mass index, young adult

## Abstract

Background/Objectives: Eating attitudes may be linked to oral health through behavioral and lifestyle mechanisms, but data on young adults remain limited. The primary objective was to assess the association between dietary attitudes and caries experience in young adults recruited from a private dental practice. Secondary analyses examined the psychometric properties of the EAT-40 and the relationships of its scores with BMI, oral health behaviors, and lifestyle factors. Methods: A cross-sectional study included 366 adults aged 18–25 years recruited by convenience sampling from a private dental practice in Iași. Eating attitudes were assessed using the EAT-40, oral health behaviors using the HU-DBI, and caries experience using the DMFT and DMFS indices. BMI was calculated based on self-reported weight and height. Exploratory factor analysis, Spearman correlations, negative binomial regression, and multiple linear regression were used. Results: The EAT-40 showed good internal consistency (α = 0.887), and the three-factor solution explained 36.6% of the variance. The total EAT-40 score showed weak positive correlations with DMFT and DMFS (ρ = 0.146; *p* = 0.005 for both), but was not significantly associated with DMFS in the fully adjusted model (IRR = 1.003; 95% CI: 0.997–1.010; *p* = 0.293) or in the sensitivity analysis (IRR = 1.005; 95% CI: 0.999–1.012; *p* = 0.122). None of the three EAT-40 dimensions was significantly associated with DMFS after adjustment. The total EAT-40 score was not significantly associated with BMI. Daily smoking was associated with higher DMFS values, whereas occasional smoking was not. Conclusions: In the study sample, EAT-40 scores were weakly associated with caries experience, but not independently with BMI. The results regarding EAT-40 dimensions remain exploratory, and the cross-sectional design does not allow for causal conclusions.

## 1. Introduction

Eating attitudes are shaped by individual, social, psychological, and environmental factors and may influence several aspects of health. These influences are particularly important in young adults, as this period involves changes in daily routine, increased autonomy in food choices, and shifts in lifestyle. During this stage, irregular meals and more frequent consumption of energy-dense foods are common, and some of these habits may persist into later adulthood [1,2,3,4].

The socioeconomic context, nutritional education, and time constraints influence both meal planning and the quality of food choices [2,5]. Busy daily schedules can favor the consumption of ready-made or fast-food products and reduce meal regularity [6,7]. Family, social groups, and the digital environment also shape food preferences and eating habits [8,9].

Body image, weight-related concerns, stress, and emotional distress have also been associated with restrictive or maladaptive eating behaviors [10,11]. Increasing professional, educational, and personal responsibilities may further disrupt eating patterns, particularly when daily schedules are irregular [12,13]. Nevertheless, substantial heterogeneity in eating patterns has been reported among young adults [14,15,16].

Eating attitudes are also related to broader lifestyle patterns. Smoking, alcohol consumption, and physical activity are commonly considered in this context because they may cluster with eating behaviors as part of broader lifestyle patterns [17,18]. Smoking has been associated with appetite regulation, weight-related concerns, and weight-control strategies [19,20]. In some situations, it may represent a means of weight control, and smoking cessation may be accompanied by fear of weight gain and changes in eating behavior [21,22,23,24,25,26]. Smoking also tends to cluster with other health-risk behaviors, including unhealthy eating and low physical activity [27,28,29].

However, lifestyle behaviors do not necessarily follow a consistent health-related pattern. For example, balanced eating may coexist with low physical activity, while smoking may coexist with otherwise health-promoting behaviors [30,31]. Food availability, access to convenience foods, economic constraints, and daily scheduling may affect several lifestyle behaviors simultaneously [5,32,33]. Social influences may further reinforce these habits during the transition to adulthood [6,34].

Eating and oral health behaviors may share common psychosocial and lifestyle determinants. Although several health-related behaviors may coexist, the relationships between them are often weak or inconsistent [18,19]. Health-promoting behaviors, particularly those related to oral hygiene, vary according to educational level, degree of health awareness, and attitudes toward prevention [35,36,37,38,39,40,41,42]. Assessing eating attitudes, oral health behaviors, and clinical dental status within the same population could clarify how these factors are related in young adults.

These inconsistencies support the simultaneous assessment of behavioral, sociodemographic, and contextual factors within the same population, as this approach may provide a more comprehensive framework for studying health-related behaviors in young adults presenting for dental services in a private dental practice [30,43]. Examining these variables within the same sample may help determine whether eating attitudes are associated with other lifestyle and oral health indicators independently of potential confounding factors. Eating disorders can affect oral health through dietary changes, recurrent vomiting, and altered salivary function. These mechanisms have been described mainly in individuals with clinically diagnosed eating disorders. The relationship between eating attitudes assessed by screening tools such as the EAT-40 and caries experience also requires clarification among young adults presenting for dental services in a private dental practice [44,45].

The main aim of the study was to assess the relationship between eating attitudes, measured using the EAT-40, and caries experience in young adults, with emphasis on the DMFS index. We also examined whether this association persisted after adjustment for demographic, anthropometric, and behavioral factors. Secondary objectives were to assess the internal consistency and factor structure of the EAT-40, examine the associations of the identified dimensions with caries experience, and analyze the relationships of the EAT-40 score with BMI, oral health behaviors, and lifestyle factors. The literature on the EAT-26 has reported solutions with three to five factors, suggesting that a structure validated in one population cannot automatically be applied to another [46,47]. In the absence of prior data on the psychometric properties of the EAT-40 in young adults from Romania, we conducted an exploratory assessment of the instrument’s internal consistency and factorial structure before examining its associations with caries experience.

We hypothesized that higher EAT-40 scores would be associated with greater caries experience and that the association between EAT-40 and DMFS would persist after adjustment for potential confounding factors. Associations between EAT-40 scores, body mass index, and the other behavioral indicators included in the study were also examined. We also explored the factorial structure of the EAT-40 and whether specific dimensions of the scale showed different associations with caries experience. For these secondary objectives, no specific hypotheses were established prior to the analysis. Because the available literature is limited and inconsistent, these analyses were considered exploratory. Their purpose was to identify potential associations and directions for future research rather than to test predefined relationships.

## 2. Materials and Methods

### 2.1. Study Design

The cross-sectional study included 366 adults aged 18 to 25, recruited through convenience sampling from a private dental office in Iași. The psychometric properties of the EAT-40 were also evaluated, together with the associations between eating attitudes, body mass index, oral health behaviors, and lifestyle factors.

The study was conducted after approval was obtained from the Ethics Committee of Grigore T. Popa University of Medicine and Pharmacy Iași (No. 221/3 August 2022). Participants were informed about the purpose of the study and the voluntary nature of participation. Data were collected and analyzed while maintaining participant anonymity and confidentiality.

### 2.2. Participants and Selection Criteria

The study was conducted between 2024 and 2026. Of the 395 individuals invited to participate, 15 declined and 14 were excluded due to incomplete data. The final sample comprised 366 participants who met the eligibility criteria and had complete data for the main analyses, representing 92.7% of those invited. Participants were recruited by convenience sampling from a private dental practice in Iași. Eligible individuals were invited to participate and were enrolled after providing voluntary informed consent. The final sample comprised all eligible participants who agreed to participate and completed the required study procedures during the recruitment period. Data were collected using self-administered questionnaires, self-reported anthropometric measurements, and a clinical dental examination.

The inclusion criteria were:

- age between 18 and 25 years;
- provision of voluntary informed consent;
- ability to understand and complete the questionnaires in Romanian;
- completion of the study questionnaires and acceptance of the clinical dental examination.

The exclusion criteria were:

- incomplete or improperly completed questionnaires;
- refusal to participate or subsequent withdrawal from the study;
- missing responses that prevented calculation of the analyzed scores;
- duplicate responses identified in the database;
- refusal to undergo the clinical dental examination.

Participants were informed about the purpose and objectives of the study, the voluntary nature of participation, and the confidentiality of the information provided. After providing informed consent, they completed the study questionnaires and underwent a clinical dental examination to assess caries experience using the DMFT and DMFS indices. Weight and height were self-reported, and body mass index was subsequently calculated from these values.

### 2.3. Measures and Assessments

#### 2.3.1. Eating Attitudes Test-40

Eating attitudes were assessed using the Eating Attitudes Test-40 (EAT-40), developed by Garner and Garfinkel to assess symptoms and concerns related to problematic eating behaviors [48]. The instrument consists of 40 items, each rated on six response options ranging from “always” to “never.” The EAT-40 is intended as a screening instrument and cannot establish a diagnosis of an eating disorder without a clinical assessment [49].

Previous studies have reported different factorial structures for instruments from the EAT family. The EAT-26 was derived from factor analysis of the original EAT-40 and included three dimensions [49], while later studies have reported different factorial solutions for the EAT-26 [46,47,50]. Because the factorial structure may vary across instruments and populations, we examined the psychometric properties and factorial structure of the EAT-40 in the present sample.

Items were scored according to the original scoring algorithm. For items scored in the symptomatic direction, the responses “always,” “usually,” and “often” were assigned 3, 2, and 1 point, respectively, whereas “sometimes,” “rarely,” and “never” were scored 0. Reverse-scored items were coded using the corresponding reversed scheme. The total score was obtained by summing all 40 items and could range from 0 to 120, with higher scores indicating more problematic eating attitudes and concerns.

The original authors proposed a cutoff score of 30 to identify individuals who may require further assessment. However, this threshold was established in the original validation sample and was not used in the present study to classify participants diagnostically. The EAT-40 score was therefore retained as a continuous variable. Categorizing participants according to a cutoff would have resulted in loss of information and reduced statistical power. In addition, the purpose of the study was to examine associations between eating attitudes and the other investigated variables rather than to estimate the prevalence of eating disorders.

The psychometric properties of the EAT-40 were assessed in the study sample. Internal consistency was examined using Cronbach’s alpha and standardized alpha. Corrected item–total correlations and changes in Cronbach’s alpha after deletion of individual items were also examined.

Exploratory factor analysis was performed using principal axis factoring with Direct Oblimin rotation. An oblique rotation was selected because the underlying dimensions of eating attitudes were expected to be potentially correlated. Sampling adequacy was assessed using the Kaiser–Meyer–Olkin index, and Bartlett’s test of sphericity was used to examine whether the correlation matrix was suitable for factor analysis.

A three-factor solution was retained based on model parsimony, the distribution of factor loadings, and the conceptual interpretability of the resulting factors. Items with absolute factor loadings below 0.30 were not assigned to the corresponding subscales. The total EAT-40 score was calculated using all 40 items, whereas scores for the three identified dimensions were obtained by summing the items with factor loadings of at least 0.30 on the respective factor.

The translation and cultural adaptation of the questionnaire followed the international recommendations for the cross-cultural adaptation of psychometric instruments [51]. Two bilingual translators (G.L., C.S.) independently translated the original questionnaire into Romanian, and the translations were then compared and merged into a consensus version. This was back-translated into English by two other independent translators (B.H., I.R.), who did not have access to the original questionnaire. The back-translations were compared with the original instrument to verify the semantic and conceptual equivalence of the items and to finalize the preliminary version in Romanian.

The preliminary version was analyzed by specialists with experience in food attitude research, who monitored the clarity of the formulations and the adequacy of the terminology. The adaptation only involved linguistic adjustments, without changing the number of items, the response options, or the way the scores were calculated.

Before collecting data for the main study, the questionnaire was pretested on 57 individuals with characteristics similar to the target population. They were asked to report any unclear or difficult-to-understand questions. Based on the observations received, minor wording adjustments were made, without changing the meaning of the original items. This stage aimed exclusively to verify the comprehensibility of the questionnaire, and the responses were not included in the statistical analyses of the study. The final version in Romanian was subsequently administered to the participants, after obtaining ethical approval.

Because this Romanian adaptation had not previously undergone independent psychometric validation, its internal consistency and factorial structure were examined exploratorily in the present study. These analyses should be regarded as preliminary psychometric evidence rather than as a formal validation of the Romanian EAT-40.

#### 2.3.2. Hiroshima University Dental Behavioral Inventory (HU-DBI)

Oral health-related behaviors and attitudes were assessed using the Hiroshima University Dental Behavioral Inventory (HU-DBI), developed by Kawamura [52]. The instrument consists of 20 statements with agree/disagree response options and is used to assess oral hygiene behaviors and attitudes toward the prevention of dental conditions.

The HU-DBI score was calculated using the 12 items included in the standard scoring system: 2, 4, 6, 8, 9, 10, 11, 12, 14, 15, 16, and 19. For items 4, 9, 11, 12, 16, and 19, an affirmative response was scored as favorable to oral health, whereas for items 2, 6, 8, 10, 14, and 15, a negative response was considered favorable.

After recoding, favorable responses were assigned a value of 1 and unfavorable responses a value of 0. The total score was calculated by summing the 12 scored items and ranged from 0 to 12, with higher scores indicating more favorable oral health behaviors and attitudes.

The Romanian-language version of the HU-DBI has previously been validated in the Romanian population and showed good reliability [53]. The reported Cronbach’s alpha coefficient was 0.816, while test–retest reproducibility was supported by an intraclass correlation coefficient (ICC) of 0.816.

#### 2.3.3. Lifestyle and Behavioral Variables

Lifestyle factors were assessed using a questionnaire covering smoking behavior, attitudes toward smoking, perceived behavioral control, intention to quit smoking, recreational activities, and alcohol consumption.

Smoking status was classified into three categories: non-smoker, occasional smoker, and daily smoker. In the multivariable analyses, non-smokers were used as the reference category.

Recreational activity was assessed using seven items covering participation in theater, cinema, clubs or discos, parties, hiking or trips, gyms, and terraces or bars. Responses were scored from 1 to 4, with 1 indicating participation once a year or less or no participation, and 4 indicating participation at least once a week. “Don’t know” responses were treated as missing. The recreational activity index was calculated as the mean of the available item scores, with higher values indicating more frequent participation.

Alcohol consumption was assessed using a single item on frequency of consumption, with five response categories: no consumption, occasional consumption, weekly consumption, consumption two to three times per week, and daily consumption. In the final sample, responses were observed only in the no-consumption, occasional-consumption, and daily-consumption categories. In the sensitivity analysis, alcohol consumption was entered as a categorical variable, with no consumption as the reference category.

The lifestyle questionnaire, which includes questions about smoking, recreational activities, and alcohol consumption, has been previously validated in the Romanian population [54]. Internal consistency was supported by a Cronbach’s alpha coefficient of 0.878, while reproducibility was supported by an intraclass correlation coefficient (ICC) of 0.830.

#### 2.3.4. Assessment of Dental Caries Experience

The DMFT (Decayed, Missing, and Filled Teeth) index was used to assess caries experience. This index provides an objective measure of cumulative caries experience at both the individual and population levels and is widely used as an epidemiological indicator [55,56].

Clinical examinations were conducted in accordance with World Health Organization recommendations for the assessment of dental caries. Examinations took place in a private dental practice using single-use examination kits and following the clinical protocol recommended for epidemiological surveys. An individual examination form was completed for each participant, recording the status of each tooth and dental surface. These records were subsequently used to calculate the DMFT and DMFS indices. Dental caries was diagnosed according to the WHO 2013 clinical criteria, and only clearly visible cavitated lesions were recorded [55].

Clinical examinations were performed by two trained and calibrated examiners. Calibration was completed before data collection to assess the consistency and reproducibility of the clinical evaluations. Inter-examiner agreement was evaluated on a common sample of participants using Cohen’s kappa coefficient. The resulting value of 0.850 indicated almost perfect agreement between the two examiners. The exact number of participants included in the calibration exercise was not separately recorded. Intra-examiner reliability was not assessed.

#### 2.3.5. Anthropometric Assessment

Body weight and height were self-reported by participants. Body mass index (BMI) was calculated as weight in kilograms divided by the square of height in meters, using the following formula:

BMI = weight (kg)/height ^2^(m^2^)

#### 2.3.6. Statistical Analysis

Statistical analyses were performed using IBM SPSS Statistics, version 26.0 (IBM Corp., Armonk, NY, USA). Categorical variables were summarized as frequencies and percentages. Numerical variables were described using means and standard deviations, as well as medians and interquartile ranges. Distributional characteristics were evaluated using skewness, the Shapiro–Wilk test, and visual inspection when appropriate.

The internal consistency of the EAT-40 and the dimensions identified through factor analysis was evaluated using Cronbach’s alpha and standardized alpha. Exploratory factor analysis was performed using principal axis factoring with Direct Oblimin rotation. Sampling adequacy was assessed using the Kaiser–Meyer–Olkin index and Bartlett’s test of sphericity. Factor loadings with absolute values below 0.30 were not considered when interpreting the factor structure.

Bivariate associations between the total EAT-40 score, the three identified dimensions, BMI, DMFT, DMFS, HU-DBI score, recreational activity index, and frequency of alcohol consumption were examined using Spearman’s rank correlation coefficient. All tests were two-tailed.

The association between the total EAT-40 score and DMFS was examined using negative binomial regression with a log link function. This model was selected because DMFS is a count variable and showed overdispersion relative to the equidispersion assumption of Poisson regression. Results were reported as incidence rate ratios (IRRs) with 95% confidence intervals.

Three sequential negative binomial regression models were fitted. Model 1 examined the unadjusted association between the total EAT-40 score and DMFS. Model 2 was adjusted for age and sex. Model 3 included age, sex, BMI, HU-DBI score, recreational activity frequency, smoking status, alcohol consumption, and socioeconomic status. These variables were selected because they were considered relevant demographic, anthropometric, behavioral, and socioeconomic factors that could influence the association between eating attitudes and caries experience. For categorical variables, male sex, non-smoker status, no alcohol consumption, and low socioeconomic status were used as reference categories.

To explore whether the EAT-40 dimensions were differently associated with caries experience, each of the three dimensions scores was entered separately into a negative binomial regression model. These models were adjusted for age, sex, BMI, HU-DBI score, and the recreational activity index.

The association between the total EAT-40 score and DMFS was further examined in a sensitivity analysis using an alternative set of covariates. This model included EAT-40 score, age, sex, smoking status, alcohol consumption, and socioeconomic status. For categorical variables, male sex, non-smoker status, no alcohol consumption, and low socioeconomic status were used as reference categories.

Factors associated with BMI were examined using multiple linear regression. The model included the total EAT-40 score, recreational activity index, HU-DBI score, age, and sex. Unstandardized regression coefficients, standardized coefficients, 95% confidence intervals, and *p*-values were reported. Multicollinearity was assessed using tolerance and variance inflation factor values, while the distribution of residuals was examined graphically.

The association between the total EAT-40 score and DMFS was considered the primary analysis. Correlation analyses and analyses involving the three EAT-40 dimensions were considered exploratory secondary analyses. To account for multiple testing in the exploratory correlation analyses, *p*-values were adjusted using the Benjamini–Hochberg false discovery rate procedure. Both unadjusted and FDR-adjusted *p*-values were considered when interpreting these results.

Statistical significance was set at *p* < 0.05.

## 3. Results

### 3.1. Sociodemographic, Anthropometric, and Behavioral Characteristics

The sample included 366 participants, with a median age of 23 years and a median BMI of 20.31 kg/m^2^. Most participants were female (67.8%), lived in urban areas (73.2%), and were of Romanian nationality (91.5%). A medium socioeconomic level was reported by 53.6% of participants. Regarding occupational status, 39.6% were employed and 31.7% were students. Daily smoking was reported by 33.3% of participants, while 70.5% reported occasional alcohol consumption (Table 1).

### 3.2. Oral Health Status

The mean DMFT index was 11.62 ± 5.38 (median 11.00, IQR = 6.00). Among its components, filled teeth had the highest mean value (6.81 ± 4.03), followed by decayed teeth (3.56 ± 3.38) and missing teeth (1.25 ± 1.55) (Table 2).

At the dental surface level, the mean DMFS index was 21.67 ± 12.34 (median 20.00, IQR = 16.00). Filled surfaces had the highest mean value (10.71 ± 9.11), followed by missing surfaces (6.28 ± 7.38) and decayed surfaces (4.68 ± 4.72) (Table 2).

### 3.3. Oral Health Attitudes and Behaviors

The mean HU-DBI (Hiroshima University Dental Behavioral Inventory) score was 6.19 ± 1.53 (median 6.00, IQR = 2.00) (Table 3). The highest proportions of scored responses were observed for the items “I often check my teeth in a mirror after brushing” (86.8%), “I put off going to the dentist until I have a toothache” (73.6%), and “I brush each of my teeth carefully” (70.5%). The lowest proportions were found for “I think I can clean my teeth without using toothpaste” (9.7%) and “I feel I sometimes take too much time to brush my teeth” (13.7%). The full distribution of scored HU-DBI responses is provided in Supplementary Table S1.

### 3.4. Psychometric Properties and Distribution of the EAT-40 Score

#### 3.4.1. Internal Consistency and Distribution of the EAT-40 Score

The EAT-40 showed good internal consistency in the overall sample (Cronbach’s α = 0.887; standardized α = 0.887). The mean total score was 45.81 ± 17.24 points (median 46.00, IQR = 24.00; range 9–95), with slight positive skewness (skewness = 0.263). When the original cutoff of 30 was considered descriptively, 297 participants (81.1%) had scores of 30 or higher, while 69 (18.9%) had scores below 30. The cutoff was not used to classify participants diagnostically.

Exploratory factor analysis identified three dimensions. Food restriction and associated distress included 19 items (mean 17.51 ± 10.92; α = 0.892), Preoccupation with food and perceived control included 7 items (mean 10.40 ± 5.66; α = 0.892), and Weight-control concerns and related behaviors included 8 items (mean 10.36 ± 5.51; α = 0.811). The total EAT-40 score was calculated using all 40 items, whereas the dimension scores included only items meeting the predefined factor-loading threshold. Corrected item–total correlations and Cronbach’s alpha values after deletion of individual items are presented in Supplementary Table S2. All three dimensions showed adequate internal consistency (Table 4).

#### 3.4.2. Exploratory Factor Analysis of the EAT-40

The adequacy of the data for factor analysis was supported by a Kaiser–Meyer–Olkin value of 0.773 and a statistically significant Bartlett’s test of sphericity (χ^2^ = 8319.776; df = 780; *p* < 0.001) (Table 5). A three-factor solution was retained, explaining 36.6% of the total variance. Interfactor correlations were weak, with absolute values ranging from 0.067 to 0.254. Six items did not reach the minimum factor-loading threshold of 0.30 on any factor and were therefore not included in the subscale scores. The complete pattern matrix for the three-factor solution is presented in Supplementary Table S3.

### 3.5. Lifestyle Behaviors and Health-Related Practices

The mean smoking attitudes score was 117.26 ± 25.14 points (median 124.00; IQR = 39.00; range 40–155), with negative skewness (skewness = −0.724), indicating that scores were more concentrated toward the higher end of the scale. The Shapiro–Wilk test showed a significant departure from normality (W = 0.940; *p* < 0.001).

The recreational activity frequency index had a mean of 2.79 ± 0.54 (median 2.84; IQR = 0.76; range 1.14–3.76), with slight negative skewness (skewness = −0.463), suggesting a modest concentration of values toward the upper part of the distribution. The Shapiro–Wilk test also indicated a significant departure from normality (W = 0.974; *p* < 0.001) (Table 6).

### 3.6. Associations Between Eating Behavior, Lifestyle, Body Mass Index, and Oral Health

Spearman correlation analysis showed weak associations between EAT-40 scores and several of the investigated variables. The total EAT-40 score was positively correlated with DMFT (ρ = 0.146, *p* = 0.005), DMFS (ρ = 0.146, *p* = 0.005), HU-DBI score (ρ = 0.110, *p* = 0.035), and recreational activity frequency (ρ = 0.114, *p* = 0.029). No significant correlation was found with BMI (ρ = −0.055, *p* = 0.291) or alcohol consumption frequency (ρ = 0.040, *p* = 0.444).

Food restriction and associated distress was positively correlated with DMFT (ρ = 0.141, *p* = 0.007), DMFS (ρ = 0.126, *p* = 0.015), HU-DBI score (ρ = 0.163, *p* = 0.002), and recreational activity frequency (ρ = 0.176, *p* = 0.001), although all correlations were weak. The Weight-control concerns and related behaviors dimension was weakly negatively correlated with BMI (ρ = −0.115, *p* = 0.028). No significant associations were found for the Preoccupation with food and perceived control dimension (Table 7).

### 3.7. Exploratory Multivariable Analyses

#### 3.7.1. Association Between EAT-40 Score and Dental Caries Experience

The association between the total EAT-40 score and the DMFS index was examined using negative binomial regression across three successive models with increasing levels of adjustment.

In the unadjusted model (Model 1), the EAT-40 total score was not significantly associated with DMFS (B = 0.004; SE = 0.0032; IRR = 1.004; 95% CI: 0.997–1.010; *p* = 0.243).

After adjustment for age and sex (Model 2), the association remained non-significant (IRR = 1.004; 95% CI: 0.998–1.010; *p* = 0.230).

In the fully adjusted model (Model 3), which included sex, age, BMI, HU-DBI score, recreational activity frequency, smoking status, alcohol consumption, and socioeconomic status, the EAT-40 total score was not significantly associated with DMFS (B = 0.005; SE = 0.0033; IRR = 1.005; 95% CI: 0.998–1.011; *p* = 0.177).

Expressed over a 10-point difference in EAT-40 score, the corresponding IRR was approximately 1.05, indicating only a small estimated change in DMFS.

Overall, no significant association between the EAT-40 total score and DMFS was found in any of the three models (Table 8).

Separate negative binomial regression models were used to examine the associations between the three EAT-40 dimensions and DMFS. Each model was adjusted for sex, age, BMI, HU-DBI score, and recreational activity frequency.

None of the three dimensions was significantly associated with DMFS after adjustment. The estimated effects were small and close to the null value: Food restriction and associated distress had an IRR of 1.002 (95% CI: 0.992–1.013; *p* = 0.651), Preoccupation with food and perceived control an IRR of 1.010 (95% CI: 0.991–1.030; *p* = 0.289), and Weight-control concerns and related behaviors an IRR of 1.009 (95% CI: 0.990–1.029; *p* = 0.366). These findings did not support an independent association between any of the three EAT-40 dimensions and DMFS (Table 9).

#### 3.7.2. Factors Associated with Body Mass Index

The multiple linear regression model examining factors associated with BMI included the total EAT-40 score, recreational activity frequency index, HU-DBI score, age, and sex. The overall model was not statistically significant (R^2^ = 0.024; adjusted R^2^ = 0.011; F(5359) = 1.792; *p* = 0.114), and the included variables explained only a small proportion of the variability in BMI.

The total EAT-40 score was not significantly associated with BMI after adjustment for the other variables in the model (B = −0.002; standardized β = −0.014; 95% CI: −0.014 to 0.011; *p* = 0.786). The HU-DBI score was positively associated with BMI (B = 0.178; β = 0.134; 95% CI: 0.040 to 0.316; *p* = 0.012), although the association was weak. Recreational activity frequency, age, and sex were not significantly associated with BMI (all *p* > 0.05) (Table 10). Because the overall model was not statistically significant and explained only 2.4% of BMI variability, these findings should be interpreted cautiously.

#### 3.7.3. Sensitivity Analysis of the Association Between EAT-40 Total Score and DMFS

In the sensitivity analysis, the EAT-40 total score was not significantly associated with DMFS after adjustment for sex, age, smoking status, alcohol consumption, and socioeconomic status (IRR = 1.005; 95% CI: 0.999–1.012; *p* = 0.122).

Daily smoking was associated with higher DMFS values compared with non-smoking (IRR = 1.528; 95% CI: 1.186–1.968; *p* = 0.001), while occasional smoking was not significantly associated with DMFS (IRR = 1.210; 95% CI: 0.888–1.648; *p* = 0.227). Occasional alcohol consumption was associated with lower DMFS values compared with no alcohol consumption (IRR = 0.646; 95% CI: 0.498–0.838; *p* = 0.001), whereas daily alcohol consumption was not significantly associated with DMFS (*p* = 0.585). No significant associations were found for sex, age, or socioeconomic status (Table 11).

These results were consistent with the main regression analyses, in which the EAT-40 total score was also not significantly associated with DMFS.

#### 3.7.4. Additional Analysis of Decayed Surfaces

As an additional analysis, the association between the EAT-40 total score and the number of decayed surfaces was examined using a fully adjusted negative binomial regression model. This analysis included 365 participants with complete data. The EAT-40 total score was not significantly associated with decayed surfaces (IRR = 1.005; 95% CI: 0.998–1.012; *p* = 0.185).

## 4. Discussion

The present study examined the relationships between eating attitudes, oral health behaviors, body mass index, and caries experience in a sample of young adults from Romania. A weak positive correlation was found between the total EAT-40 score and DMFS. However, the EAT-40 total score was not significantly associated with DMFS in the negative binomial regression models, including after adjustment for the other variables considered in the study. The same result was observed in the sensitivity analysis. Previous studies have reported associations between eating disorders or disturbed eating behaviors and poorer oral health outcomes [44,45], although the present findings suggest that the relationship with caries experience may be limited when other factors are taken into account.

When the EAT-40 dimensions were examined separately, none of the three dimensions was significantly associated with DMFS after adjustment for the other variables included in the models. Food restriction and associated distress showed weak positive correlations with both DMFT and DMFS in the bivariate analysis, but these associations did not remain significant in the adjusted regression models. The weak bivariate associations may reflect dietary characteristics or other factors common to eating attitudes and caries experience. However, the available data do not allow the temporal order or direct effect of eating attitudes on caries occurrence to be established. The analysis of the EAT-40 dimensions was exploratory, and these findings should therefore be interpreted cautiously and confirmed in other populations.

The EAT-40 and the three dimensions identified in the present sample showed good internal consistency. Correlations between the factors were relatively weak. The three factors explained 36.6% of the total variance, and six items did not reach the factor-loading threshold of 0.30. Although the identified dimensions showed acceptable internal consistency, these findings do not establish their validity and should be interpreted as exploratory. The interpretation of the scores and associations of each subscale therefore remains exploratory and requires confirmation in an independent sample. Health-related behaviors in student populations are often heterogeneous and may be influenced by several contextual factors [57,58]. Previous studies have reported different factorial structures for EAT instruments, suggesting that their dimensional structure may vary according to the population and cultural context in which they are used [46,47,50].

A weak positive correlation was found between the HU-DBI score and the Food restriction and associated distress dimension. Given the small magnitude of the correlation, this finding should not be interpreted as evidence of a strong relationship between the two constructs. It may indicate that, in some participants, greater concerns related to eating coexist with greater attention to oral health behaviors. Health-related behaviors do not necessarily occur in a consistent favorable or unfavorable pattern, and different combinations of behaviors may be present within the same individual [18,19,31].

In the multivariable model for BMI, the total EAT-40 score was not independently associated with BMI, which was consistent with the absence of a significant correlation in the bivariate analysis. Recreational activity frequency was also not significantly associated with BMI. The HU-DBI score showed a weak positive association with BMI, although the overall regression model was not statistically significant.

The index assessed the frequency of participation in recreational activities, without measuring the level of physical exertion. For this reason, the results for this variable should be interpreted in relation to the activities included in the questionnaire, not to physical activity in general.

No significant association between the EAT-40 score and BMI was observed in either the bivariate or adjusted analysis. Another possible explanation is related to the perception of one’s own weight and dietary behaviors. Weight dissatisfaction may be accompanied by higher EAT-40 scores and attempts at dietary restriction, whose relationships with BMI are difficult to capture in a cross-sectional study. However, these explanations remain hypotheses. The model explained only 2.4% of the variation in BMI, showing that most of this variation was not captured by the factors analyzed. In this context, the lack of a significant association cannot be considered evidence of the absence of a relationship between eating attitudes and BMI. Smoking status may also be related to body-weight patterns, and previous population-based data have shown differences in adiposity according to smoking status [59].

Tobacco use remains an important public health concern [17,18,19,60]. In the sensitivity analysis, daily smokers had higher expected DMFS values than non-smokers, whereas occasional smoking was not significantly associated with DMFS. This finding is in line with previous reports showing that smoking often occurs together with other health-risk behaviors [17,18,19,29]. No significant association was found between sex and DMFS. Occasional alcohol consumption was associated with lower DMFS values, while daily alcohol consumption was not significantly associated with DMFS. This result should be interpreted cautiously, particularly because only four participants reported daily alcohol consumption. Therefore, the corresponding regression estimate is imprecise and was not interpreted substantively. It should not be interpreted as evidence of a protective effect of alcohol, since the observed association may be related to residual confounding or to the uneven distribution of participants across the alcohol-consumption categories.

The relationships between eating attitudes, oral health behaviors, and the other lifestyle indicators were generally weak. Most statistically significant correlations were small, with coefficients ranging approximately from 0.11 to 0.18. This pattern suggests that the variables examined did not form a single, consistent behavioral profile. Similar findings have been reported in studies examining the clustering and coexistence of health-related behaviors [18,19,30,31]. The sample size may also have contributed to some very small associations reaching statistical significance. Statistical significance should therefore be considered together with the magnitude and potential clinical relevance of the observed associations.

The weak correlations between eating attitudes and caries experience did not remain significant in the adjusted regression models. This suggests that the observed relationships may be influenced by other factors included in the analyses. At this stage, eating behaviors may be better considered as part of a broader lifestyle assessment rather than using the EAT-40 to estimate caries risk. A preventive approach that considers dietary behaviors, smoking, and oral health practices together may be relevant for young adults [31,40].

### 4.1. Study Limitations

The present study has several limitations. First, its cross-sectional design does not permit causal inferences regarding the relationships between eating attitudes, oral health behaviors, lifestyle factors, and caries experience. The associations observed therefore reflect relationships within the study sample and do not establish their direction.

Smoking, alcohol consumption, and eating attitudes were assessed by self-report, which may introduce social desirability bias. Participants may have underreported behaviors perceived as socially disapproved, thereby influencing the observed associations. The direction and magnitude of this bias could not be assessed in the study. Weight and height were self-reported, not measured in the study. Some may have misremembered their values or reported a lower weight or a higher height. Therefore, the calculated BMI may contain errors, which could have influenced the observed associations. We cannot assess how large these errors were, as we do not have direct measurements to compare the self-reported values with.

Participants were between 18 and 25 years of age, resulting in a relatively narrow age range. This may have limited the ability to detect age-related differences and restricts the generalizability of the findings to other age groups. In addition, women represented approximately two-thirds of the sample, which should be considered when interpreting differences according to sex.

Participants were recruited through convenience sampling from a single private dental practice. Individuals who attend such a service may have different characteristics from those of the general population, including oral health and access to care. This selection limits the generalizability of the results to all young adults.

Several psychological factors that may influence eating attitudes, including body image perception, stress, emotional distress, and specific motivations for weight control, were not directly assessed. Future studies including these variables may help clarify their role in the relationship between eating attitudes and oral health.

Although the total EAT-40 scale and the three identified dimensions showed good internal consistency, the three-factor structure identified in this study was exploratory and should be confirmed in independent samples of young adults from Romania.

The bivariate correlations between EAT-40 scores and DMFS were statistically significant, but the correlations were weak and did not remain significant in the multivariable regression models. Given the sample size, small correlations may still reach statistical significance, so their clinical relevance should be interpreted with caution. The multivariable model for BMI also explained only a small proportion of BMI variability, indicating that most of the variation in BMI was not accounted for by the variables included in the model.

Recreational activity was assessed according to the frequency of participation in different activities rather than by using a standardized measure of physical activity, intensity, or energy expenditure. The resulting index should therefore be interpreted as a general measure of participation in recreational activities and not as a direct indicator of physical activity level.

DMFT and DMFS reflect cumulative caries experience, including previous disease and treatment, whereas the EAT-40 assesses current eating attitudes. Because of this difference in timing, the cross-sectional findings should not be interpreted as showing that current eating attitudes contributed to caries accumulated in previous years. In the additional analysis using decayed surfaces as the outcome, the EAT-40 total score was not significantly associated with untreated caries.

The caries experience of people who attend dental services may differ from that of people who do not. In addition, the exclusive inclusion of participants with complete data may introduce selection bias if the excluded individuals differ from those analyzed.

Future studies using longitudinal designs, more diverse samples, and a broader assessment of psychological and behavioral factors may help clarify the direction and possible mechanisms underlying the relationships observed in this study.

### 4.2. Future Research Directions

The results of this study indicate that further research is needed to examine the relationships between eating attitudes, lifestyle, and oral health in young adults. Future studies should include more diverse samples to determine whether the associations observed in the present study are also found in other population groups.

Further research could also include variables such as stress level, body image perception, emotional distress, and specific weight-control behaviors. These factors may help explain some of the variation in EAT-40 scores and provide a more detailed understanding of their relationship with oral health. Examining individual lifestyle components separately may also help identify which behaviors are more closely associated with eating attitudes or oral health behaviors.

Given the association observed between smoking and caries experience, greater attention should also be given to the way in which different health-risk behaviors coexist. Longitudinal studies, conducted in more diverse samples and including a broader assessment of psychological and behavioral factors, would allow eating attitudes, lifestyle behaviors, and oral health to be followed over time. Such studies may help clarify the direction of the observed associations and the mechanisms involved.

Future research could also include objective measurements of weight and height, as well as assessments of body composition, to provide a more precise evaluation of the relationships between eating attitudes, lifestyle, and nutritional status.

The present findings may also provide a basis for the development of health education programs for young adults. Approaches that address diet, oral health, smoking, and other lifestyle behaviors together may be more appropriate than interventions focused on a single behavior.

## 5. Conclusions

As this is a cross-sectional study, we cannot determine whether eating attitudes preceded carious lesions or whether there is a causal relationship between them. Among young adults recruited by convenience sampling from a private dental practice, EAT-40 scores showed weak positive correlations with DMFT and DMFS, but these associations were not significant in the adjusted regression models. The EAT-40 showed good internal consistency in the study group, while the three-factor structure remains exploratory and requires confirmation in an independent sample.

None of the three EAT-40 dimensions was significantly associated with DMFS after adjustment. The associations between EAT-40 scores, BMI, HU-DBI, and the other lifestyle indicators were generally weak. The total EAT-40 score was not significantly associated with BMI in either the bivariate or multivariable analysis.

In the sensitivity analysis, daily smoking was associated with higher DMFS values, whereas occasional smoking was not significantly associated with DMFS. Occasional alcohol consumption was associated with lower DMFS values. However, this finding should be interpreted cautiously and cannot be considered evidence of a protective effect of alcohol.

Overall, the observed associations were small and restricted to this convenience sample, and the results do not support the use of eating attitudes as a predictor of caries risk.

## Figures and Tables

**Table 1:** Sociodemographic, Anthropometric, and Behavioral Characteristics of Participants.

Characteristic	Total Sample (n = 366)
Age, years, median (IQR)	23.00 (1.00)
Height, cm, mean ± SD	171.20 ± 7.10
Weight, kg, median (IQR)	60.00 (13.00)
BMI, kg/m^2^, median (IQR)	20.31 (2.10)
Sex, n (%)	
Male	118 (32.2)
Female	248 (67.8)
Residential area, n (%)	
Urban	268 (73.2)
Rural	44 (12.0)
Peri-urban	54 (14.8)
Nationality, n (%)	
Romanian	335 (91.5)
Other	31 (8.5)
Socioeconomic level, n (%)	
Low	70 (19.1)
Medium	196 (53.6)
High	100 (27.3)
Occupational status, n (%)	
Unemployed	20 (5.5)
Student	116 (31.7)
Self-employed	85 (23.2)
Employed	145 (39.6)
Smoking status, n (%)	
Never	182 (49.7)
Occasionally	62 (16.9)
Daily	122 (33.3)
Alcohol consumption, n (%)	
Never	104 (28.4)
Occasionally	258 (70.5)
Daily	4 (1.1)

**Table 2:** Dental Caries Experience and Its Components.

Indicator	Mean ± SD (n = 366)	Median (IQR) n = 366
DMFT index	11.62 ± 5.38	11.00 (6.00)
Decayed teeth	3.56 ± 3.38	3.00 (4.00)
Missing teeth	1.25 ± 1.55	1.00 (2.00)
Filled teeth	6.81 ± 4.03	6.00 (5.00)
DMFS index	21.67 ± 12.34	20.00 (16.00)
Decayed surfaces	4.68 ± 4.72	3.00 (5.00)
Missing surfaces	6.28 ± 7.38	5.00 (10.00)
Filled surfaces	10.71 ± 9.11	8.00 (7.00)

**Table 3:** Distribution of the HU-DBI Score.

Indicator	Mean ± SD	Median (IQR)	Range
HU-DBI score	6.19 ± 1.53	6.00 (2.00)	1–10

Note: The theoretical HU-DBI score ranges from 0 to 12, with higher scores indicating more favorable oral health attitudes and behaviors. HU-DBI, Hiroshima University Dental Behavioral Inventory; IQR, interquartile range; SD, standard deviation.

**Table 4:** Internal Consistency and Distribution of the EAT-40 Total and Factor Scores.

Scale/Factor	Cronbach’s Alpha	Std Alpha	Mean ± SD	Median (IQR)	Range	Skewness
EAT-40 total scale	0.887	0.887	45.81 ± 17.24	46.00 (24.00)	9–95	0.263
Food restriction and associated distress	0.892	0.895	17.51 ± 10.92	16.00 (12.00)	0–50	0.657
Preoccupation with food and perceived control	0.892	0.893	10.40 ± 5.66	10.00 (7.00)	0–21	0.135
Weight-control concerns and related behaviors	0.811	0.778	10.36 ± 5.51	10.00 (8.00)	0–21	0.099

Note: EAT-40, Eating Attitudes Test-40; IQR, interquartile range; SD, standard deviation.

**Table 5:** Summary of Exploratory Factor Analysis Results for the EAT-40.

KMO	Bartlett’s χ^2^	df	*p*-Value	Extraction Method	Rotation Method	Retained Factors	Variance Explained	Factor Correlation Range	Items with Loadings < 0.30
0.773	8319.776	780	<0.001	Principal axis factoring	Direct Oblimin	3	36.6%	−0.254 to 0.162	6

Note: KMO, Kaiser–Meyer–Olkin measure of sampling adequacy.

**Table 6:** Lifestyle and Health-Related Behavioral Indicators.

Variable	Mean ± SD	Median (IQR)	Range	Skewness	Shapiro–Wilk
Smoking-related attitudes score	117.26 ± 25.14	124.00 (39.00)	40–155	−0.724	W = 0.940; *p* < 0.001
Recreational activity frequency index	2.79 ± 0.54	2.84 (0.76)	1.14–3.76	−0.463	W = 0.974; *p* < 0.001

**Table 7:** Correlations of EAT-40 Score with Body Mass Index, Oral Health, and Lifestyle Indicators.

Variable	EAT Total	Food Restriction and Associated Distress	Preoccupation with Food and Perceived Control	Weight-Control Concerns and Related Behaviors
BMI	−0.055 (0.291; q = 0.466)	−0.007 (0.891; q = 0.949)	−0.028 (0.594; q = 0.750)	−0.115 (0.028; q = 0.087)
DMFT	0.146 (0.005; q = 0.030) *	0.141 (0.007; q = 0.034) *	0.092 (0.079; q = 0.177)	0.009 (0.856; q = 0.949)
DMFS	0.146 (0.005; q = 0.030) *	0.126 (0.015; q = 0.060)	0.091 (0.081; q = 0.177)	0.071 (0.174; q = 0.298)
HU-DBI	0.110 (0.035; q = 0.093)	0.163 (0.002; q = 0.024) *	0.0 (0.997; 1.0 q = 0.997)	0.029 (0.575; q = 0.750)
Recreational activity frequency index	0.114 (0.029; q = 0.087)	0.176 (0.001; q = 0.024) *	−0.077 (0.140; q = 0.258)	0.082 (0.116; q = 0.232)
Alcohol consumption frequency	0.040 (0.444; q = 0.627)	0.048 (0.360; q = 0.540)	−0.012 (0.822; q = 0.949)	−0.006 (0.909; q = 0.949)

Note. Values are Spearman’s correlation coefficients (unadjusted *p*-values; Benjamini–Hochberg FDR-adjusted q-values). * Indicates statistical significance after FDR adjustment (q < 0.05).

**Table 8:** Negative Binomial Regression Models for the Association Between EAT-40 Total Score and DMFS.

Model	Adjustment	B	SE	IRR [Exp(B)]	95% CI for IRR	*p*-Value
Model 1	Unadjusted	0.004	0.0032	1.004	0.997–1.010	0.243
Model 2	Adjusted for sex and age	0.004	0.0032	1.004	0.998–1.010	0.230
Model 3	Adjusted for sex, age, BMI, HU-DBI score, recreational activity frequency, smoking status, alcohol consumption, and socioeconomic status	0.005	0.0033	1.005	0.998–1.011	0.177

**Table 9:** Adjusted Negative Binomial Regression Models for EAT-40 Dimensions and DMFS.

EAT-40 Dimension	B	SE	IRR [Exp(B)]	95% CI for IRR	*p*-Value
Food restriction and associated distress	0.002	0.0054	1.002	0.992–1.013	0.651
Preoccupation with food and perceived control	0.010	0.0098	1.010	0.991–1.030	0.289
Weight-control concerns and related behaviors	0.009	0.0099	1.009	0.990–1.029	0.366

Note: Each model was adjusted for sex, age, BMI, HU-DBI score, and recreational activity frequency. IRR, incidence rate ratio; CI, confidence interval; SE, standard error.

**Table 10:** Multiple Linear Regression Analysis of Factors Associated with Body Mass Index.

Predictor	B	SE	Standardized β	95% CI for B	*p*-Value
EAT-40 total score	−0.002	0.006	−0.014	−0.014 to 0.011	0.786
Recreational activity frequency index	−0.204	0.231	−0.047	−0.659 to 0.251	0.378
HU-DBI score	0.178	0.070	0.134	0.040 to 0.316	0.012
Age	−0.036	0.037	−0.052	−0.110 to 0.038	0.337
Sex	0.087	0.244	0.020	−0.392 to 0.567	0.720

Model summary: R^2^ = 0.024, Adjusted R^2^ = 0.011, F(5359) = 1.792, *p* = 0.114.

**Table 11:** Sensitivity Analysis Results for the Association Between EAT-40 Total Score and DMFS.

Covariate	IRR [Exp(B)]	95% CI	*p*-Value
EAT-40 total score	1.005	0.999–1.012	0.122
Sex (female vs. male) *	1.244	0.972–1.593	0.083
Age	0.998	0.961–1.035	0.905
Daily smoking vs. never *	1.528	1.186–1.968	0.001
Occasional smoking vs. never *	1.210	0.888–1.648	0.227
Daily alcohol consumption vs. none *	1.329	0.479–3.683	0.585
Occasional alcohol consumption vs. none *	0.646	0.498–0.838	0.001
High socioeconomic level vs. low *	1.230	0.892–1.696	0.206
Medium socioeconomic level vs. low *	1.189	0.893–1.581	0.235

Note: IRR, incidence rate ratio; CI, confidence interval. Reference categories were male sex, never smoking, no alcohol consumption, and low socioeconomic level. * Indicates comparisons with the reference categories specified above.

## Data Availability

The data supporting the findings of this study are available from the corresponding author upon reasonable request, due to confidentiality considerations.

## Supplementary Materials

The following supporting information can be downloaded at https://www.mdpi.com/article/10.3390/nu18193273/s1, Supplementary Table S1. Distribution of Scored Responses to the HU-DBI Items; Supplementary Table S2. Corrected Item–Total Correlations and Cronbach’s Alpha if Item Deleted for the EAT-40; Supplementary Table S3. Pattern Matrix for the Three-Factor EAT-40 Solution.

## Abbreviations

The following abbreviations are used in this manuscript:

EAT	Eating Attitudes Test
HUDBI	Hiroshima University Dental Behavioral Inventory
IQR	Interquartile Range
BMI	Body Mass Index
SPSS	Statistical Package for the Social Sciences
ICC	Intraclass Correlation Coefficient
IRR	Incidence Rate Ratio
SD	Standard Deviation
CI	Confidence Interval
*p*	*p*-value
β	standardized regression coefficient
SE	Standard Error
DMFT	Decayed, Missing and Filled Teeth
DMFS	Decayed, Missing and Filled Surfaces
