# Supplementary material for: Association Between Eating Attitudes and Dental Caries Experience in Young Adults Attending a Private Dental Practice: A Cross-Sectional Study
Source: Nutrients. 2026 Oct 5;18(19):3273. doi: 10.3390/nu18193273 (PMC13666866; doi:10.3390/nu18193273)
Supplement: Supplementary file 1 [file nutrients-18-03273-s001.zip › nutrients-4585289-supplementary.pdf]

Suplemmentary Table S1. Distribution of Scored Responses to the HU-DBI Items

HU-DBI item	Scored response. n (%)
My gums tend to bleed when I brush my teeth	70 (30.8)
I have noticed some white sticky deposits on my teeth	151 (66.5)
I think that I cannot help having false teeth when I am old	108 (47.6)
I think my teeth are getting worse despite my daily brushing	56 (24.7)
I brush each of my teeth carefully	160 (70.5)
I have never been professionally taught how to brush	146 (64.3)
I think I can clean my teeth without using toothpaste	22 (9.7)
I often check my teeth in a mirror after brushing	197 (86.8)
It is impossible to prevent gum disease with toothbrushing alone	135 (59.5)
I put off going to the dentist until I have a toothache	167 (73.6)
I have used a dye to see how clean my teeth are	158 (69.6)
I feel I sometimes take too much time to brush my teeth	31 (13.7)

Supplementary Table S2. Corrected Item–Total Correlations and Cronbach's Alpha if Item Deleted for the EAT-40

	Scale Mean if Item Deleted	Scale Variance if Item Deleted	Corrected Item-Total Correlation	Squared Multiple Correlation	Cronbach's Alpha if Item Deleted
Like eating with other people	44.0044	290.323	.197	.248	.885
Prepare foods for others but do not eat what I cook	45.5947	283.357	.457	.627	.881
Become anxious prior to eating.	45.5947	283.870	.426	.569	.882
Am terrified about being overweight.	45.0661	289.965	.194	.467	.885
Avoid eating when I am hungry.	45.6344	279.383	.560	.703	.879
Find myself preoccupied with food.	44.9251	280.822	.478	.736	.881
Have gone on eating binges where I feel that I may not be able to stop.	45.4978	284.331	.429	.682	.882
Cut my food into small pieces.	45.1101	282.523	.405	.645	.882
Aware of the calorie content of foods that I eat	44.7533	283.558	.363	.673	.883
Particularly avoid food with a high carbohydrate content (i.e. bread. rice. potatoes. etc.	45.1013	280.260	.438	.596	.881
Feel boated after meals	45.1454	286.523	.301	.414	.884

Feel that others would prefer if I ate more.	45.5419	282.320	.459	.645	.881
Vomit after I have eaten	45.4714	295.002	.059	.188	.887
Feel extremely guilty after eating.	45.6872	281.065	.637	.807	.879
Am preoccupied with a desire to be thinner	44.6960	284.699	.346	.793	.883
Exercise strenuously to burn off calories.	44.8458	283.193	.381	.649	.882
Weigh myself several times a day.	45.5463	273.930	.638	.658	.877
Like my clothes to fit tightly.	45.3084	282.090	.405	.555	.882
Enjoy eating meat	44.5639	289.026	.230	.646	.885
Wake up early in the morning	44.8458	281.786	.440	.735	.881
Eat the same foods day after day	45.1850	282.355	.466	.524	.881
Think about burning up calories when I exercise.	45.0176	278.619	.483	.591	.880
Have regular menstrual periods.	44.7930	282.908	.395	.414	.882
Other people think that I am too thin	45.3568	280.965	.435	.580	.881
Am preoccupied with the thought of having fat on my body.	44.9912	276.929	.542	.700	.879
Take longer than others to eat my meals	44.7930	281.545	.453	.781	.881
Enjoy eating at restaurants.	44.5815	286.457	.285	.527	.884
Avoid foods with sugar in them	45.3524	278.256	.558	.638	.879
Eat diet foods	45.4846	276.640	.591	.701	.879
Feel that food controls my life.	44.9868	282.402	.404	.751	.882
Display self-control around food.	44.7753	283.095	.390	.756	.882
Feel that others pressure me to eat.	45.4626	280.719	.470	.688	.881
Give too much time and thought to food	45.5991	285.949	.400	.622	.882
Feel uncomfortable after eating sweets	45.4405	282.796	.432	.646	.881
Engage in dieting behavior.	44.9648	276.733	.566	.816	.879
Enjoy trying new rich foods.	44.6960	283.505	.362	.695	.883
Take laxatives	45.6828	296.368	.047	.427	.886

Suffer from constipation	45.7269	296.244	.090	.362	.885
Have the impulse to vomit after meals	45.4581	296.010	.022	.242	.889
Like my stomach to be empty	45.2599	298.636	-.050	.215	.891

Supplementary Table S3. Pattern Matrix for the Three-Factor EAT-40 Solution.

	Factor		
	1	2	3
Feel extremely guilty after eating.	.846		
Prepare foods for others but do not eat what I cook	.691		
Am preoccupied with the thought of having fat on my body.	.686		
Avoid eating when I am hungry.	.680		
Weigh myself several times a day.	.631		
Feel that others would prefer if I ate more.	.631		
Eat diet foods	.623		
Feel that others pressure me to eat.	.615		
Feel uncomfortable after eating sweets	.547		
Particularly avoid food with a high carbohydrate content (i.e. bread. rice. potatoes. etc.	.544		
Become anxious prior to eating.	.538		
Have gone on eating binges where I feel that I may not be able to stop.	.498		
Give too much time and thought to food	.488		
Eat the same foods day after day	.482		
Avoid foods with sugar in them	.480		
Other people think that I am too thin	.445		
Feel boated after meals	.411		
Cut my food into small pieces.	.388		
Am terrified about being overweight.	.373		
Like my clothes to fit tightly.			
Like eating with other people			
Enjoy eating at restaurants.			
Take longer than others to eat my meals		-.855	
Engage in dieting behavior.		-.817	
Feel that food controls my life.		-.816	
Find myself preoccupied with food.		-.815	
Enjoy trying new rich foods.		-.740	
Enjoy eating meat		-.716	
Have regular menstrual periods.		-.423	
Like my stomach to be empty			
Vomit after I have eaten			
Am preoccupied with a desire to be thinner			.856
Display self-control around food.			.814
Wake up early in the morning			.778
Aware of the calorie content of foods that I eat			.738

Exercise strenuously to burn off calories.	.647
Think about burning up calories when I exercise.	.571
Take laxatives	.350
Suffer from constipation	
Have the impulse to vomit after meals	
